# Prenatal arsenic exposure induces immunometabolic alteration and renal injury in rats

**DOI:** 10.3389/fmed.2022.1045692

**Published:** 2023-01-11

**Authors:** Radha Dutt Singh, Ratnakar Tiwari, Vineeta Sharma, Hafizurrahman Khan, Siddhartha Gangopadhyay, Sukhveer Singh, Kavita Koshta, Shagun Shukla, Nidhi Arjaria, Kapil Mandrah, Pankaj Ramji Jagdale, Satyakam Patnaik, Somendu Kumar Roy, Dhirendra Singh, Ashok Kumar Giri, Vikas Srivastava

**Affiliations:** ^1^Systems Toxicology and Health Risk Assessment Group, CSIR-Indian Institute of Toxicology Research, Lucknow, Uttar Pradesh, India; ^2^Academy of Scientific and Innovative Research, New Delhi, India; ^3^Department of Biotechnology, Faculty of Engineering and Technology, Manav Rachna International Institute of Research and Studies, Faridabad, Haryana, India; ^4^Advanced Imaging Facility, CSIR-Indian Institute of Toxicology Research, Lucknow, Uttar Pradesh, India; ^5^Regulatory Toxicology Group, CSIR-Indian Institute of Toxicology Research, Lucknow, Uttar Pradesh, India; ^6^Molecular and Human Genetics Division, CSIR-Indian Institute of Chemical Biology, Kolkata, West Bengal, India

**Keywords:** prenatal, immunometabolism, metabolic impairment, hypermethylation, nephropathy and chronic kidney disease

## Abstract

Arsenic (As) exposure is progressively associated with chronic kidney disease (CKD), a leading public health concern present worldwide. The adverse effect of As exposure on the kidneys of people living in As endemic areas have not been extensively studied. Furthermore, the impact of only prenatal exposure to As on the progression of CKD also has not been fully characterized. In the present study, we examined the effect of prenatal exposure to low doses of As 0.04 and 0.4 mg/kg body weight (0.04 and 0.4 ppm, respectively) on the progression of CKD in male offspring using a Wistar rat model. Interestingly, only prenatal As exposure was sufficient to elevate the expression of profibrotic (TGF-β1) and proinflammatory (IL-1α, MIP-2α, RANTES, and TNF-α) cytokines at 2-day, 12- and 38-week time points in the exposed progeny. Further, alteration in adipogenic factors (ghrelin, leptin, and glucagon) was also observed in 12- and 38-week old male offspring prenatally exposed to As. An altered level of these factors coincides with impaired glucose metabolism and homeostasis accompanied by progressive kidney damage. We observed a significant increase in the deposition of extracellular matrix components and glomerular and tubular damage in the kidneys of 38-week-old male offspring prenatally exposed to As. Furthermore, the overexpression of TGF-β1 in kidneys corresponds with hypermethylation of the TGF-β1 gene-body, indicating a possible involvement of prenatal As exposure-driven epigenetic modulations of TGF-β1 expression. Our study provides evidence that prenatal As exposure to males can adversely affect the immunometabolism of offspring which can promote kidney damage later in life.

## Introduction

Chronic kidney disease (CKD) incidence is increasing at an alarming rate. More than 10% of the world’s population suffers from some form of kidney disease (1). The rate is as high as 17% in India (2). Increased blood pressure, high blood sugar levels, adiposity, and abnormal cholesterol or triglyceride levels are common risk factors for CKD (3, 4). However, up to 40% of CKD cases are due to genetic factors and unknown causes, which may include or are related to environmental pollutants such as heavy metals, pesticides, nanomaterials, air pollutants, and several commonly used drugs, including painkillers and immunosuppressants (5). Exposure to environmental pollutants is also associated with metabolic and cardiovascular diseases (6, 7), which may further aggravate the prevalence of kidney disease.

Arsenic (As) is among the major environmental pollutants, affecting > 500 million people worldwide with exposure levels above the WHO maximum permissible limit of 10 ppb (8). Contaminated drinking water from natural geological sources is the most common source of As exposure, which increases the risk of multiorgan cancers, cardiovascular disease, metabolic syndrome, and renal disorders (9, 10). However, the effect of early-life exposure to low As concentrations (around the permissible limit of 10 μg/L) on human health has not been extensively studied, although evidence suggests that type of exposure affects human health (11). The New Hampshire Birth Cohort Study (NHBCS) began in 2009 with the purpose of studying how arsenic, affects the health of pregnant mothers and their babies (12). The study included women who had been exposed to low to moderate quantities of arsenic through the use of private wells as well as dietary sources. Analyses of NHBCS data have indicated links between prenatal arsenic exposure and fetal and neonatal development (12, 13), infant infection rates (14), immunological profiles, inflammatory markers, and leptin levels in cord blood (15–17), and gene expression and DNA methylation in the fetal placenta (18–22). As a result, it is becoming increasingly clear that even low-to-moderate amounts of arsenic have various impacts on the developing fetus and result in poor infant health outcomes. Prenatal exposure to arsenic and other heavy metals has been shown to be nephrotoxic (23). A Chilean study found a rise in CKD mortality in young adults after *in utero* and childhood exposure to arsenic through drinking water (24). Previous studies suggested that As exposure during early life as well as during the adult stage affects the metabolic and physiological pathways of the body and promotes diabetes and obesity (11). Studies have also shown increased body-mass index (BMI) in As exposed individuals (25). The metabolic and physiological effect of As varies at different dose thresholds (26, 27). This non-monotonic As-induced physiological and metabolic dysregulation has been observed, which is primarily due to the complex interaction of inorganic As with different nuclear receptors (26, 28, 29).

Arsenic (As) is known to affect multiple organs, including the kidney (10), which is an important site for As uptake and accumulation (30). As is being increasingly associated with kidney toxicity in As affected regions of South East Asia, Taiwan, and several western countries (31–37). Kidney injury caused by chronic As exposure is characterized by hypercalciuria, albuminuria, proteinuria, nephrocalcinosis, β-2 microglobulin, and renal injury at the cellular and subcellular level (38–40). As can induce kidney cell proliferation and cause changes in cell fate and function (41). Arsenic has also been known to play a synergistic role in amplifying glycogen nephrosis in diabetic rats (42). Population studies have found a latency pattern of increased kidney cancer mortality that lasted for at least 25 years after high exposure levels began to drop (43). Studies have also shown an inverse relationship between urinary As levels and CKD (34). However, most of these As studies are either adult exposure or *in vitro* studies. Further studies on As exposure also suggest that it could cross the transplacental barrier (44) and might affect the developing fetus (45, 46). Studies have shown that early life As exposure (85 ppm) promotes renal injury (47). However, the association between As low to moderate-dose prenatal exposure (27, 48) and its role in adult-onset CKD has not been characterized.

Previous studies have also shown that As exposure modulates the expression of several profibrotic (TGF-β1) and proinflammatory genes, including TNF-α, RANTES, IL-1α, MIP-2α, and MIP-3α (49–51). Some of these cytokines are collectively classified as adipokines (TGF-β1 and TNF-α) and are known to play a significant role in the development and progression of diabetes (52, 53). The cumulative effect of these changes may lead to metabolic dysregulation and, in the long run, may promote diabetic nephropathy. However, most of these studies show the effect of prolonged As exposure at higher doses. They, therefore, cannot delineate which phase of life (gestational, pubertal, or adult) is most susceptible to As exposure. Due to chronic As exposure in humans, it is impossible to assess prenatal exposure’s contribution in isolation, which is the highly vulnerable stage of development. Therefore, we used the Wistar rat as an animal model for an isolated *in utero* As exposure experiment and investigated whether only prenatal exposure to low-to-moderate environmentally reported levels of As (27, 48) could cause adult-onset kidney disease. To achieve this, we exposed female rats to As prior to mating and continued till the gestation period, and monitored the ensuing pups till late adulthood for signs of metabolic changes and kidney disorders. We also studied progressive alteration in the levels of inflammatory, adipogenic, and metabolic parameters. We further studied DNA methylation changes in the gene-body of TGF-β1, which might be a probable cause of metabolic dysregulation and kidney injury. Our study, thus, tries to investigate whether only *in utero* As exposure is sufficient to imprint lifelong changes that may promote CKD and related disorders in the progeny born.

## Materials and methods

### Materials

Sodium (meta) arsenite (NaAsO_2_) (≥90% pure), Periodic Acid-Schiff (PAS) Kit, and Trichrome Stain (Masson) Kit were procured from Sigma-Aldrich, St. Louis, MO, United States. High-capacity cDNA reverse transcription kits and Qubit dsDNA BR Assay Kit were purchased from Thermo Fisher Scientific, Eugene, OR, United States. Milliplex TGF-β1 Magnetic Bead Single Plex Kit, Milliplex MAP Rat Cytokine/Chemokine Magnetic Bead Panel, Milliplex Rat Kidney Toxicity Magnetic Bead Panel 1 were purchased from EMD Millipore, Billerica, MA, United States. Bio-Plex Pro™ Rat Cytokine Assay and Bio-Plex Pro™ Rat Diabetes Assay kits were bought from Biorad, Laboratories, Hercules, CA, United States. Anti-NPHS2, anti-TGF-β1, and anti-Fibronectin were purchased from Abcam, United States. Anti-Glucagon Alexa Fluor 570 and anti-Insulin Alexa Fluor 488 were purchased from eBioscience, Inc., San Diego, CA, United States. Secondary antibodies such as Goat anti-rabbit IgG (H + L) Cross-Adsorbed Secondary Antibody, Alexa Fluor 488 (A-11008), and Goat anti-Mouse IgG (H + L) Cross-Adsorbed Secondary Antibody, Alexa Fluor 594 (A-11005) were brought from Thermo Fisher Scientific, Eugene, OR, United States. Reagents such as paraformaldehyde (PFA), D-glucose (dextrose), NaCl, creatinine, sodium hydroxide (NaOH), picric acid, Hematoxylin, Eosin Y, glutaraldehyde (TEM grade), sodium cacodylate, Heparin sodium, Tris, EDTA, Tween 20, citric acid, DNase I, Fluoroshield DAPI, and Bovine serum albumin (BSA) were bought from Sigma-Aldrich, St. Louis, MO, United States. Pierce™ BCA Protein Assay Kit was purchased from Thermo Fisher Scientific, Eugene, OR, United States. Hydrochloric acid (HCl) and paraffin wax and DPX were brought from SRL Pvt. Ltd., Maharashtra, India. Insulin (biphasic isophane insulin) was procured from Gland Pharma Limited, Hyderabad, India. Phosphate buffer saline (1X) and Trizol were brought from Thermo Fisher Scientific, Eugene, OR, United States. Specific primers (Supplementary Table 1) for the quantitative real-time PCR and Methylated DNA Immunoprecipitation (MeDIP) assay were purchased from Integrated DNA Technologies, Inc., Coralville, IA, United States. SYBR Premix Ex Taq II (TlIRNase H Plus) was procured from Takara Bio USA, Inc., San Jose, CA, United States.

### Animal experiment

Animal experimental protocols were approved by the Institutional animal ethics committee (IAEC) of CSIR-Indian Institute of Toxicology Research, India (Reference No.: IITR/IAEC/14/14; Year: 2014). Specific-pathogen-free female and male Wistar rats aged 6 weeks old were obtained from the animal housing facility of the approving institution. Animals were kept in polypropylene cages under standard laboratory conditions of temperature 25 ± 5°C, relative humidity 50 ± 15% and dark/light period of 12:12 h. The animals were fed on a standard pellet diet (Complete pellet diet, Provimi Kliba, Switzerland) and sterile distilled water as drinking water [with As level below the limit of detection (LOD), i.e., 1 ng/L] *ad libitum*.

Female rats were randomly grouped into three groups, with 10 female rats in each group. They were treated with sterile distilled water (control) or As doses (As 0.04 ppm and As 0.4 ppm) for 15 days prior to mating and continued throughout the gestation period. NaAsO_2_ dose was prepared in sterile distilled water and administered at 0.04 (within the Benchmark dose range of 40–60 μg/L of As in drinking water) and 0.4 mg/kg body weight doses per day (0.04 and 0.4 ppm, respectively) (27). The dose was administered daily *via* oral gavage consistently at the same time (forenoon) throughout the treatment in a dose volume of 10 mL/kg under normal feeding conditions. After 15 days of As treatment to females, the male and female rats were kept for breeding in a 2:1 (female: male) ratio. The dosing of female rats was continued during the mating period. The mating was confirmed by a smear test. When 70% of female rats were positive, the male and female rats were separated. The treatment was continued throughout the gestation period.

Five prenatally exposed male pups (one male pup per dam) from each group born to As exposed mothers were sacrificed on postnatal day (PD) 2 during the forenoon. Whole kidney and plasma samples were collected and stored at −80°C for further processing. The remaining pups were kept with their mother. After PD 21 days, the animals were weaned, and male and female pups were segregated. All experiments were conducted on male pups due to their stable hormonal and physiological parameters, as well as males’ higher susceptibility to As.

Bodyweight of all animals was measured at 2, 4, 6, 8, 12, 16, 20, 24, 28, 32, 36, and 38 weeks before sacrificing. In addition, random blood glucose level was determined in 12- and 38 weeks prenatally exposed male offspring during the first hour of the daylight cycle.

At 12 weeks of age (PD 12-weeks), five male rats (one male pup per dam) from each group were sacrificed, and plasma and whole kidneys were collected during the forenoon, snap-frozen in liquid nitrogen, and stored at −80°C for further processing. Similarly, at PD 38 weeks, five males (one male pup per dam) from the mentioned groups were again sacrificed, and their plasma was collected, snap-frozen, and kept at −80°C for further processing. The whole kidney and pancreas were also taken and fixed in 4% PFA for immunohistochemistry (IHC) and histopathology. Tissue and plasma samples were collected in the forenoon from non-fasting animals prenatally exposed to As.

Freshly voided urine samples were also collected from five prenatally exposed male rats (one male pup per dam) from each group 1 h after the start of the light cycle at PD 12- and 38-week time points. The urine samples were collected from prenatally exposed offspring with normal feeding conditions. The animals were taken from the cages and placed in a petri dish for collection. The urine sample collected was stored at −80°C for further processing.

### Measurement of cytokine, adipokine, and metabolic biomarker levels in blood plasma

To detect cytokine and adipokine levels in the blood plasma of prenatally exposed offspring *via* Multiplex bead-based assay, the Bio-Plex MAGPIX Multiplex Reader platform (Bio-Rad Laboratories, Hercules, CA, United States) was used. The level of cytokines and adipokines such as TGF-β1, IL-1α, MIP-2α, MIP-3α, RANTES, TNF-α, and VEGF was determined using Milliplex MAP TGF-β1 Single Plex Magnetic Bead Kit, Milliplex MAP Rat Cytokine/Chemokine Magnetic Bead Panel and Bio-Plex Pro™ Rat Cytokine Assay Kit. Similarly, the level of metabolic biomarkers such as ghrelin, leptin, and glucagon was measured using the Bio-Plex Pro™ Rat Diabetes Assay kit. For the detection of different analytes, blood plasma samples stored at −80°C used. The level of glucagon in the pancreatic tissue lysate was also accessed using the Bio-Plex Pro™ Rat Diabetes Assay kit. The samples were thawed, centrifuged, and processed as per the manufacturer’s instructions and protocols. The results were analyzed using the Bio-Plex Manager software (Bio-Rad Laboratories). Results were expressed as mean fluorescent intensity (MFI).

### Oral glucose tolerance test and intraperitoneal insulin tolerance test

Oral glucose tolerance test and IPITT were performed at 12-, 16-, 20, and 24-week time points on 5 *in utero* exposed and control male rats. OGTT was done at 9:00 a.m. after overnight fasting of 16 h in all the groups simultaneously and on the same day. OGTT was performed using 2.0 g/kg body weight D-glucose (dextrose), prepared in sterile physiological saline (0.85% NaCl) solution at 37°C. The overnight fasted animals received a glucose challenge by oral gavage. Basal blood glucose level and blood glucose level at 0 h, 15 min, 30 min, 45 min, 1 h, 1.5 h, and 2 h were measured in the blood from the tail vein using a portable glucometer (AccuChek Active, India). Between the OGTT and IPITT, there was a gap of 1 week for the animals to gain their unstressed normal condition. For IPITT, animals were pre-fasted for 6 h and were challenged with 1 U/Kg Insulin (biphasic isophane insulin) diluted in a sterile physiological saline solution. The glucose level was then measured in the blood from the tail vein at the same time points using a portable glucometer as mentioned above (for OGTT).

### Assessment of kidney injury parameters

The kidney injury parameters such as early kidney toxicity biomarker Kidney injury molecule-1 (KIM-1), serum-creatinine level, and urinary protein level were assessed. Expression of KIM-1 was evaluated by using a Milliplex kidney toxicity biomarker panel on the bead-based Multiplex assay platform Bio-Plex MAGPIX Multiplex Reader. In 2-day-old pups, KIM-1 level was assessed in the kidney tissue lysate, whereas in the 12- and 38-week animals, urine samples were used to determine KIM-1 level. Tissue lysate and urine samples stored at −80°C were thawed, centrifuged, and processed as per the manufacturer’s instructions and protocols. Results are expressed as mean fluorescent intensity (MFI).

Total urinary protein was determined using Pierce™ BCA Protein Assay Kit following the manufacturer’s protocol and was read on Spectra Max MS Multimode Microplate reader (Molecular Devices, LLC., San Jose, CA, United States). Urinary total protein result was expressed relative to urinary creatinine level.

Furthermore, creatinine level in plasma was measured by Jaffe’s reaction (1886) method (54). Approximately 1 g/L creatinine stock was prepared in 0.4 M HCl. Working creatinine standards of 0.2, 0.4, 0.6, 0.8, 1.0, 1.2, 2.5, and 5.0 mg/dl concentrations were prepared in 0.4 M HCl. Alkaline picrate was freshly prepared by mixing 0.5 M sodium hydroxide (NaOH) and saturated picric acid in a 1:1 ratio. A total of 10 μl of working standards and plasma samples of respective groups were added to the 96-well microplate. To it, 120 μl of water and 20 μl of freshly prepared alkaline picrate were added and incubated at 25°C for 45 min. After incubation, absorbance was measured at 505 nm on a Spectra Max MS Multimode Microplate reader. The standard graph was plotted between the known concentration and their respective optical density (OD). The concentration of the samples was obtained by plotting the value of unknown concentration on the standard plot. The concentration of creatinine was expressed in mg/dl.

### Histopathological and ultrastructural analysis

Kidneys at a 38-week time point from five prenatally exposed male rats (one male rat per litter) from each group were isolated during forenoon and fixed in 4% PFA, embedded in paraffin wax, and 5 μm thick sections were cut for analysis after staining. To assess the histopathological changes, Hematoxylin and Eosin (H&E), Trichrome, and PAS staining were done.

The sections were stained with H&E according to the standard H&E protocol (55). Briefly, the deparaffinized, rehydrated tissue sections were stained with Mayer’s Hematoxylin for 5–8 min, washed, and counterstained with Eosin Y. The stained slide was washed, dehydrated, mounted in DPX, and visualized under the light microscope (LeicaOrthoplan). Similarly, for Trichrome and PAS staining, the sections were stained with Trichrome and PAS stain using Trichrome Stain (Masson) and Periodic Acid-Schiff (PAS) Kits as per manufacturers’ instructions and visualized under the light microscope (Leitz, Germany).

Five sections per animal and five animals per group were taken for the histopathological analysis. Random images were taken per section and were analyzed by two independent pathologists, and they were unaware of the study and treatment status of the 38-weeks old prenatally exposed animals. To determine the glomerular surface area, urinary space, and PAS-positive area, 10 random images were chosen per section for the analysis using Image J software (National Institutes of Health, Bethesda, MD, USA). The mean of the glomerular surface area and urinary space determined was calculated for each animal and plotted on a graph. The slides were visualized under a light microscope (Leitz, Germany).

To study the effects of prenatal As exposure on the cortical region of the kidney tissue (proximal tubular, distal tubular, and glomerular region), ultrastructural analysis of the kidney sections was carried out by Transmission Electron Microscopy (TEM). Animals were perfused with 100 mL of ice-cold saline (0.9% NaCl in deionized water with 10 U/ml heparin; pH 7.3–7.4) and fixed in 4% PFA (pH 7.3–7.4) containing 0.2% glutaraldehyde (TEM grade) and 0.1 M sodium cacodylate. Tissues were cut into small cube-shaped slices (0.5–1.0 mm thick) and further processed for TEM analysis, as mentioned in our previous article (56).

### Immunohistochemistry

Similar to histopathological analysis, five sections per animal and five animals per group were taken and imaged randomly for immunohistochemistry. The kidney tissue sections from the PD 38-week time point were deparaffinized, rehydrated, and processed for IHC. The sections were kept in Tris-EDTA antigen retrieval buffer (Tris-EDTA: 10 mM Tris,1 mM EDTA, 0.05% Tween-20, pH 9.0 or Citrate buffer: 10 mM Citric acid, 0.05% Tween 20, pH 6) at 98°C for 20 min for antigen retrieval. The sections were then washed in Phosphate Buffer Saline (PBS) with 0.1% Tween-20 (PBST, pH 7.6) followed by 1 h blocking process in 3% Bovine serum albumin (BSA) and then incubated overnight with primary antibody at 4°C (anti-NPHS2, anti-TGF-β1, anti-Fibronectin, Anti-Glucagon Alexa Fluor 570, and anti-insulin Alexa Fluor 488). The sections were then washed and incubated with primary antibodies (anti-NPHS2, anti-TGF-β1, and anti-Fibronectin) and with Alexa Fluor labeled secondary antibody [Goat anti-rabbit IgG (H + L) Cross-Adsorbed Secondary Antibody, Alexa Fluor 488, A-11008; and Goat anti-Mouse IgG (H + L) Cross-Adsorbed Secondary Antibody, Alexa Fluor 594, A-11005] at room temperature (RT). All sections were then mounted with the Fluoroshield DAPI and visualized under Leica TCS SPE confocal microscope (Leica Microsystems, Nussloch, Germany).

### Quantitative real time gene expression profile

Total RNA was extracted from the kidney by the Trizol method (57), followed by quantification, DNase digestion, and cDNA preparation using High-Capacity cDNA Reverse Transcription Kit and assessed by quantitative real-time PCR by using gene-specific primers (Supplementary Table 1) and SYBR Premix Ex Taq II (TlIRNase H Plus). QuantStudio™ 6 Flex Real-Time PCR System (Life Technologies, Eugene, OR, United States) was used for the quantitative real-time PCR. β-actin and 18S RNA were used as the internal control. Threshold values (Ct) were used to calculate relative gene expression using the ΔΔCt method (58).

### Methylated DNA immunoprecipitation assay

CpG methylation enrichment was determined by the MeDIP assay. The MeDIP assay was performed as described in our previous manuscript (41). The enriched and isolated DNA was subjected to quantitative real-time PCR by using specific primers (forward: GACTCTCCACCTGCAAGACC, reverse: CCTCAGAGCTCACCGTTGTT), using QuantStudio™ 6 Flex Real-Time PCR System with SYBR Green as a fluorescent reporter using SYBR Premix Ex Taq II (TliRNase H Plus). Results were expressed as fold enrichment as compared to isotype controls.

### Statistical analysis

All studies contain at least three different breeding rounds per assay, with at least *n* = 5 (one pup per litter) per assay. Data reported in this study (ELISA, IHC, real-time PCR, MeDIP, and ChIP) are the minimum mean of three biological replicates. Descriptive statistical analysis was performed to determine the coefficient of variance (CoV) and standard error mean (SEM). All data are presented in the graphs as mean ± SEM, and a *p* < 0.05 was set for statistical significance. To analyze multiple groups, a one-way analysis of variance (ANOVA) analysis with a confidence interval (CI) of 95% was done. To check the normality of the data, the Kolmogorov–Smirnov test was performed. Analysis was done using GraphPad software (GraphPad Software, v. 6.0; San Diego, CA, USA).

## Results

### Sex ratio of pups (fraction male) born to mothers

In the present study, the sex ratio of pups born to pregnant mothers was not significant and unusually very low (Supplementary Figure 1).

### Prenatal As exposure induces the expression of profibrotic and proinflammatory cytokines

**FIGURE 1:**
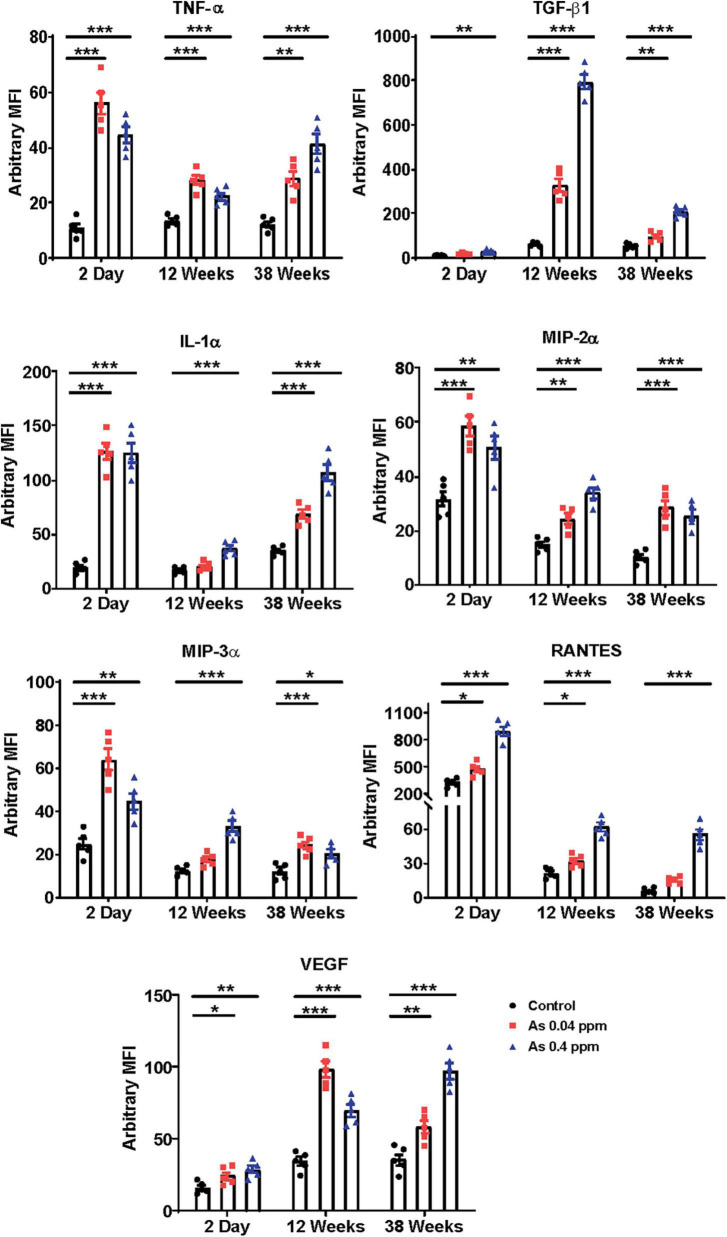
Prenatal As exposure promotes the expression of proinflammatory cytokines and adipokine. Prenatal As exposure induces expression of proinflammatory cytokine and adipokines in the progeny. The level of cytokines and adipokines was assayed in blood plasma at 2 days, 12- and 38 weeks. The data is represented as mean fluorescence intensity (MFI). The data represent the mean ± SEM, *n* = 5 (number of experimental sets). *p < 0.05, **p < 0.001, and ***p < 0.0001.

The level of chemokines and proinflammatory cytokines was assessed in prenatally exposed offspring at 2-day, 12- and 38 weeks of age (Figure 1). The level of TNF-α and profibrotic (TGF-β1) cytokine was determined in the blood plasma samples at different time points. The level of TNF-α was significantly higher in the blood plasma samples of 0.04 ppm (*p* ≤ 0.0001; CoV 15.9%) and 0.4 ppm (*p* ≤ 0.001; CoV 14.3%) prenatal As exposed 2-day old pups as compared to the time-point control (CoV 29.85%), which was observed till 12- (CoV 13.33, 13.06 and 13.51% in control, 0.04 and 0.4 ppm exposed groups, respectively) and 38 weeks (CoV 19.57, 20.45 and 18.91% in control, 0.04 and 0.4 ppm exposed groups, respectively) post-prenatal As exposure (*p* ≤ 0.001). TGF-β1, a potent profibrotic biomarker, was also assessed in plasma samples of prenatally As exposed offspring at different time points. The level of TGF-β1 was significantly upregulated (*p* ≤ 0.05) in plasma samples of 2-day-old pups (CoV 16.72, 17.04 and 17.99% in 0.04 and 0.4 ppm exposed groups, respectively), which persisted in 12- (CoV 13.95, 19.2 and 8.669% in control, 0.04 and 0.4 ppm exposed groups, respectively) and 38-weeks (CoV 19.35, 18.95 and 11.75% in control, 0.04 and 0.4 ppm exposed groups, respectively) old prenatally exposed groups as compared to the time-point control. A positive correlation was observed between the TGF-β1 expression and prenatal As treatment dose. A significant increase in the level of IL-1α, MIP-2α, MIP-3α, RANTES, and VEGF was observed at 2-day which persisted till 12 and 38 weeks of age in prenatally As exposed offspring. The coefficient of variance of all the timepoints and all the groups has been shown in Supplementary Table 2. In 12- and 38-week-old prenatally exposed female offspring, the level of GMCSF, IL-7, RANTES, MIP-3α, VEGF, IL-1β, and TGF- β1 cytokines were also found to be significantly overexpressed (Supplementary Figure 2).

### Alteration in glucose homeostasis

**FIGURE 2:**
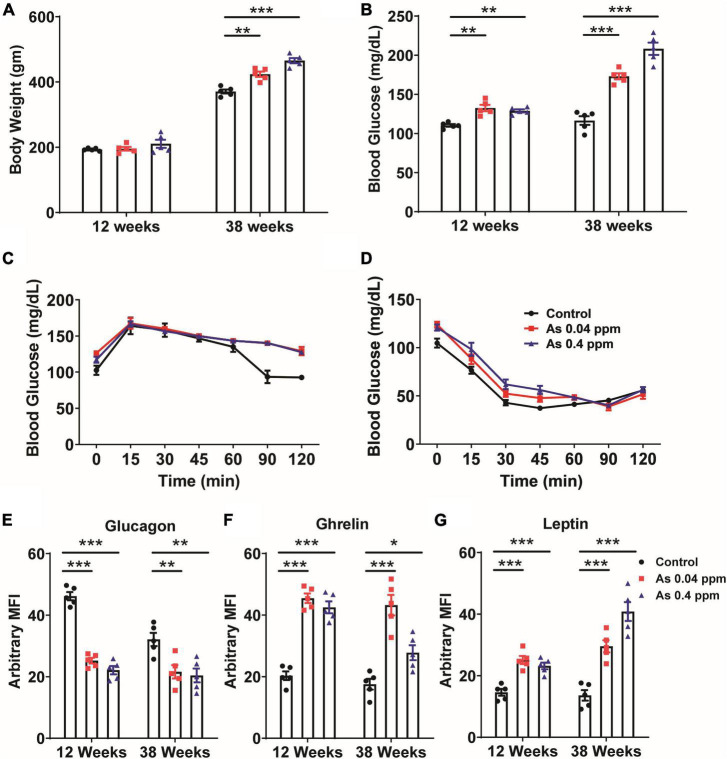
Prenatal As exposure promoted weight gain, dysregulated glucose metabolism, and induced the expression of metabolic markers. Body weight **(A)** and blood glucose level **(B)** at 12 and 38 weeks of age. OGTT **(C)** and IPITT **(D)** at a 24-week time point. The levels of glucagon **(E)**, ghrelin **(F)**, and leptin **(G)** were determined in blood at 12 and 38 weeks of age. The data represent the mean ± SEM, *n* = 5 (number of experimental sets). *p < 0.05, **p < 0.001, and ***p < 0.0001.

Furthermore, we investigated the effect of prenatal As exposure on glucose metabolism. We examined body weight, blood glucose level, and metabolic markers. Bodyweight was determined at different time points. However, no significant change in body weight was observed till 20 weeks of age (Figure 2A; Supplementary Figure 3). At 38 weeks, a significant change in the body weight was observed in a dose-dependent manner (Figure 2A; Supplementary Figure 3).

The random blood glucose level was determined at 12- and 38 weeks of age in prenatally exposed offspring. The mean blood glucose level was 122 ± 1.655, 133 ± 4.057, and 128 ± 1.934 mg/dl in control, 0.04 and 0.4 ppm prenatally As exposed groups, respectively, at 12 weeks of age (Figure 2B). At 38 weeks of age, the mean blood glucose level was 172 ± 3.860 (0.04 ppm) and 175 ± 7.794 mg/dl (0.4 ppm) in As exposed groups as compared to 127.5 ± 5.582 mg/dl in the control group (Figure 2B).

We also performed OGTT and IPITT in 12-, 16-, 20-, and 24-week old prenatally exposed rats. There was no significant change in glucose tolerance till 20 weeks of age. However, we observed a significant change in glucose tolerance in the prenatally exposed rats at 24 weeks of age. Following OGTT, while the control groups were able to restore their blood glucose level at 2 h following the initial increase, the As exposed groups could not regain their normal blood glucose even after 2 h of oral glucose administration (Figure 2C). However, following IP insulin administration (IPITT), no significant difference in glucose levels was observed in any group (Figure 2D).

### Assessment of metabolic and adipogenic factors

**FIGURE 3:**
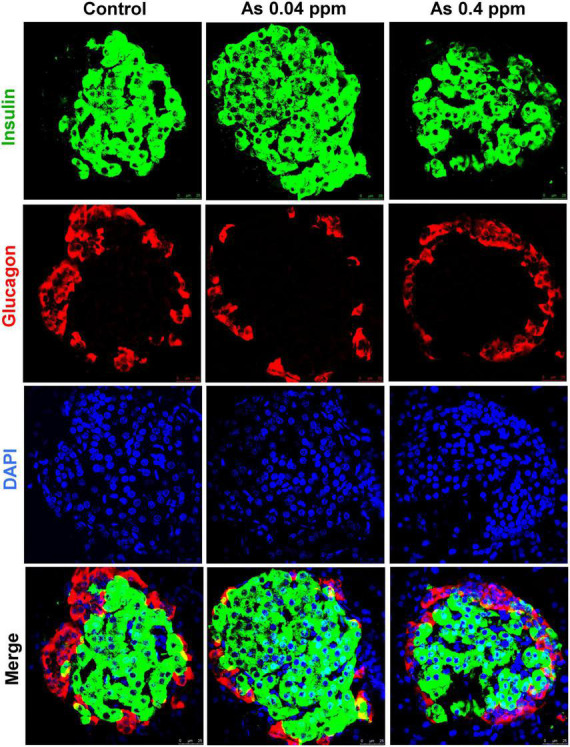
Effect of prenatal As exposure on the pancreas. The levels of insulin and glucagon were also determined through the IHC of the pancreas isolated from 38 weeks old progeny.

Change in the level of metabolic and adipogenic factors was later assessed. We examined glucagon, ghrelin, and leptin levels in the blood plasma of 12- and 38-weeks rats who were prenatally exposed to As (Figures 2E–G). A decrease in blood glucagon was observed in 12- and 38-week-old prenatally exposed progeny. The change in glucagon level was highly significant (*p* ≤ 0.0001) in 12-weeks old As exposed animals as compared with the controls (Figure 2E). The level of ghrelin was significantly higher in 12- and 38-week-old prenatally As exposed progeny (0.04 and 0.4 ppm prenatally exposed rats) compared with their respective controls (Figure 2F). Change in leptin level was also observed in 12- and 38-weeks old As exposed progeny. The leptin level in the blood was significantly higher at both 12 and 38 weeks of age (Figure 2G). A decrease in the glucagon level in plasma was further validated by Immunohistochemical staining (IHC) in the pancreas (Figure 3). The number of glucagon-positive cells was lesser than the controls at 38 weeks (Figure 3) and correlated with decreased circulating glucagon level (Figures 2E–G). Moreover, the number of insulin-positive cells was also lower in prenatal As exposed groups at 0.4 ppm (Figure 3). The level of glucagon was also observed in the pancreatic tissue lysate and interestingly, the level of glucagon was significantly decreased in 12 and 38 weeks old prenatally arsenic-exposed rats (Supplementary Figure 6).

### Prenatally As exposed animals show higher levels of nephrotoxicity markers

**FIGURE 4:**
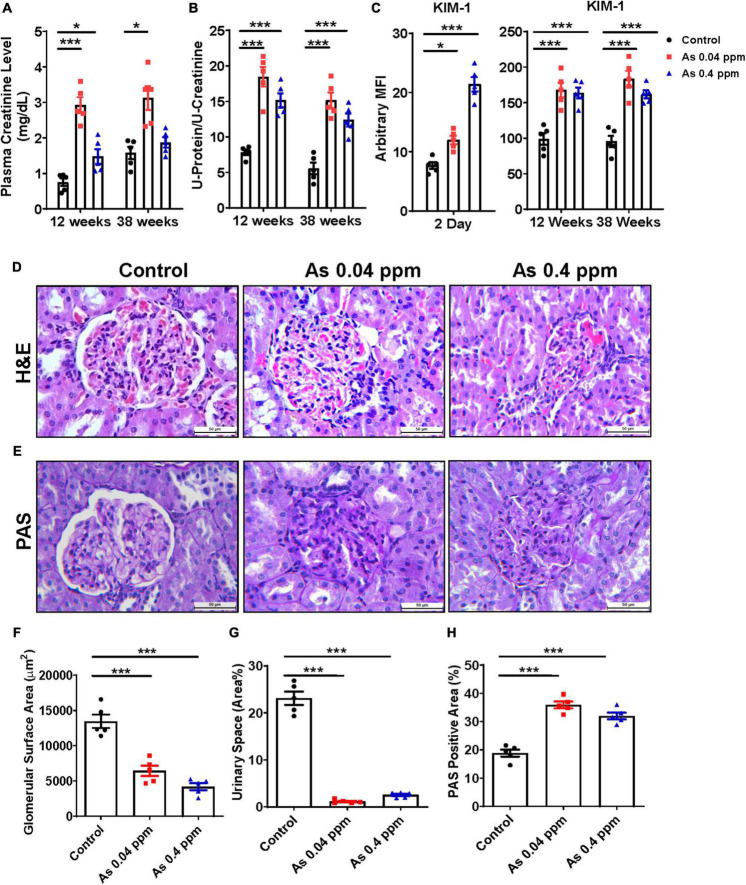
Prenatal As exposure elevates renal toxicity markers and associated cytokines in exposed rat offspring. The renal toxicity markers were assessed in prenatally exposed animals. Plasma creatinine level **(A)** and urinary total protein **(B)** at 12- and 38-week time points. Early kidney toxicity biomarker KIM-1 was assessed in kidney tissues at 2 days and in urine samples at 12- and 38 weeks of age **(C)**. H&E **(D)** and PAS staining **(E)** of kidney sections of 38-week-old prenatally As exposed rats were done. A significant loss in the glomerular surface area in As treated groups was observed which is also represented graphically **(F)**. Loss in urinary space was also calculated and represented graphically **(G)**. The mesangial expansion was also calculated based on the level of PAS-positive material **(H)**. The kidney toxicity biomarker and cytokine data are represented as mean fluorescence intensity (MFI). The bar shown in the histopathological images represents a 50 μm distance. The data represent the mean ± SEM, *n* = 5 (number of experimental sets). Values were compared with their respective controls. *p < 0.05 and ***p < 0.0001.

Plasma creatinine levels were significantly higher in the animals prenatally exposed to 0.04 and 0.4 ppm As as compared to controls at 12 and 38 weeks of age except in the 0.4 ppm group at 38 weeks (Figure 4A). Similarly, higher urinary protein levels were found in 12 and 38 weeks (Figure 4B) As exposed animals were both at 0.04 ppm and 0.4 ppm doses. In addition, higher creatinine and protein levels correspond with an increase in KIM-1 expression, an early biomarker of renal injury (Figure 4C). There was significantly higher expression of KIM-1 in 0.04 ppm and 0.4 ppm prenatally As exposed 2-day-old pups (Figure 4C). Further increase in the KIM-1 expression was also observed at both 12- and 38-weeks (Figure 4C) in 0.04 and 0.4 ppm exposed groups.

### Structural alterations in the glomerular region of the kidney after prenatal As exposure

The glomerulus is an important part of the kidney where the majority of blood is filtered and is essential for the kidney’s proper function. Members of the chemokine superfamily and proinflammatory genes are associated with glomerular injury (59). There were structural aberrations in the glomerular region, including reduced glomerular area and urinary space, less open capillaries, mesangial cell expansion, and increased cellularity as indicated by H&E staining in the As exposed progeny (Figure 4D). The images were analyzed through Image J software, and the glomerular surface area (Figure 4F), urinary space, and mesangial expansion (Figure 4G) are represented graphically. The glomerular surface area was reduced to half in some cases, while there was a drastic decrease in urinary space in As exposed groups compared to controls.

We also assessed the deposition of PAS-positive material in the kidney by qualitative estimation with periodic acid-Schiff (PAS) staining. An increased level of PAS-positive material was observed in the glomeruli and interstitium of the kidney of As exposed group (Figure 4E). We quantified the % area of PAS-positive material by Image J software, and the analyzed data was graphically represented, which clearly shows significant and considerable deposition of PAS-positive material. The kidney of prenatal As exposed progeny exhibited 10–15% more area with PAS-positive material (Figure 4H).

### Ultrastructural alterations in the kidney of as exposed animals

**FIGURE 5:**
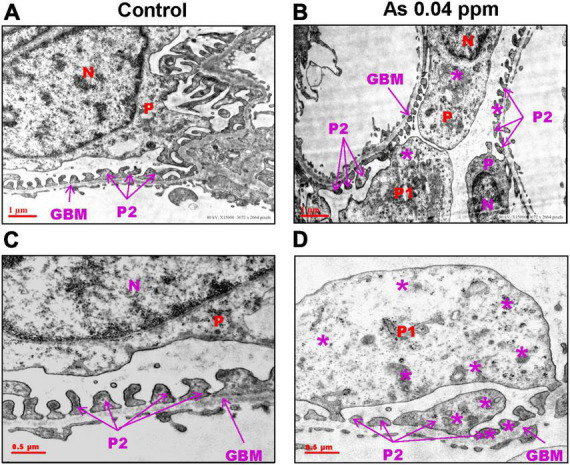
Prenatal As exposure causes ultrastructural changes in the glomerulus which is reflected in the abnormal expression pattern of podocin (NPHS2). Prenatal As exposure leads to swollen, extended (P1), and damaged foot processes (P2) **(B–D)** as compared with the control **(A–C)** as observed in the TEM sections of kidney isolated from 38-week-old offspring. The podocytes (P) extend branching foot processes (P1 and P2) which lie around the glomerular basement membrane (GBM). The asterisk represents the site of damage in P1 and P2 **(A–D)**.

**FIGURE 6:**
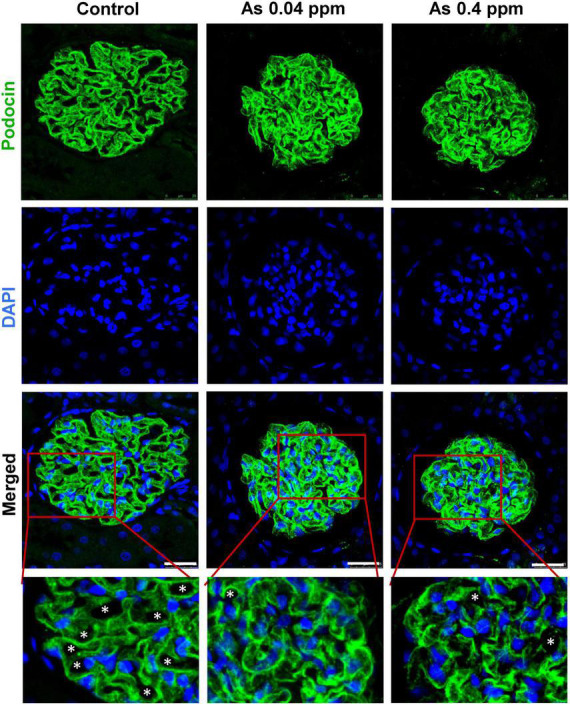
Prenatal As exposure induced ultrastructural changes in the glomerulus are reflected in the abnormal expression pattern of podocin (NPHS2). IHC images of the glomerular region showing staining for podocin and DAPI. The expression of podocin is continuous in the control kidney sections, while it is diffused and discontinuous in As exposed offspring. Inset views of corresponding images clearly show the pattern of podocin expression in the control and treated section. Asterisk represents open tubules which are reduced in As exposed animals. Bar = 25 μm.

Kidneys were further examined for pathological changes at the ultrastructural level through transmission electron microscopy (TEM). Distinct cellular and subcellular changes were found in prenatally exposed progeny kidneys. There was a decrease in the number of podocytes, effacement of their foot processes, and overall mesangial expansion suggesting glomerular injury (Figures 5B, D) compared to their respective controls (Figures 5A, C). Swollen podocytes were observed in prenatally As exposed progeny compared with their respective control groups. The foot processes of podocytes in prenatally As exposed offspring were either swollen (P1) or shorter (P2) (Figures 5B, D) as compared with their respective controls (Figures 5A, C). The damage in the podocyte foot process was also reflected in the expression pattern of the Podocin (NPHS2) protein (Figure 6). The expression of Podocin was diffused and discontinuous with a lack of open tubules in the As exposed progeny compared to the control (Figure 6). Diffuse and discontinuous staining of podocytes indicates the probability of altered glomerular filtration.

**FIGURE 7:**
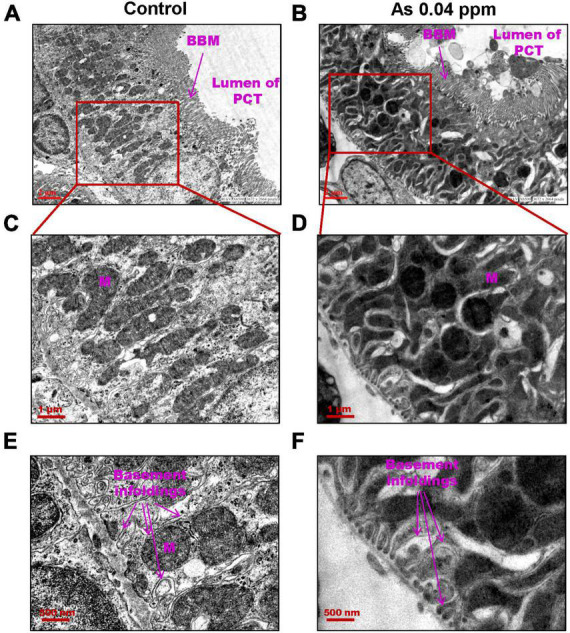
Prenatal As exposure leads to ultrastructural damage in PCT. TEM analysis showed increased mitochondrial damage. The damage in the mitochondrial membrane and its cristae are prominent in kidneys isolated from 38 weeks old prenatally As exposed animals. Furthermore, damage in basement infoldings was also observed **(C–F)**. The brush border membranes (BBM) are also damaged in exposed groups **(A–B)**.

**FIGURE 8:**
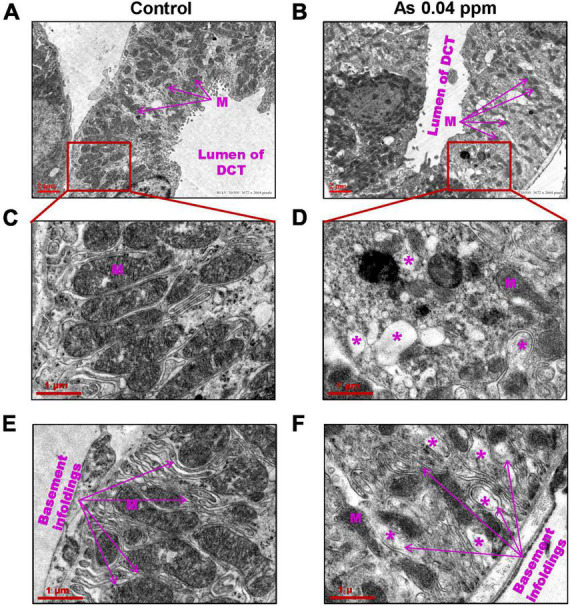
Prenatal As exposure leads to ultrastructural damages in DCT. The TEM analysis showed damage to the mitochondrial membrane and its cristae. The damaged mitochondria (M) **(A–D)** and damaged basement membrane infoldings were prominent in 38 weeks old prenatally As exposed animals **(E–F)**. The asterisk shows the site of damage.

Furthermore, ultrastructural changes in the proximal convoluted tubules (PCT) (Figure 7) and distal convoluted tubules (DCT) (Figure 8) were observed in prenatally exposed progeny. There was severe damage in mitochondria in PCT (Figure 7) and DCT (Figure 8) of prenatally As exposed progeny. Membranes and cristae of mitochondria were not distinct, and the demarcation of membranes of the basement infoldings was altered in both PCT (Figure 7) and DCT (Figure 8). There was significant damage to the brush border membrane (BBM) of the PCT of the kidney at certain places. The mitochondria had completely lost their internal content in some instances, especially in the DCT (Figures 7D, F).

### Assessment of the renal matrix proteins

**FIGURE 9:**
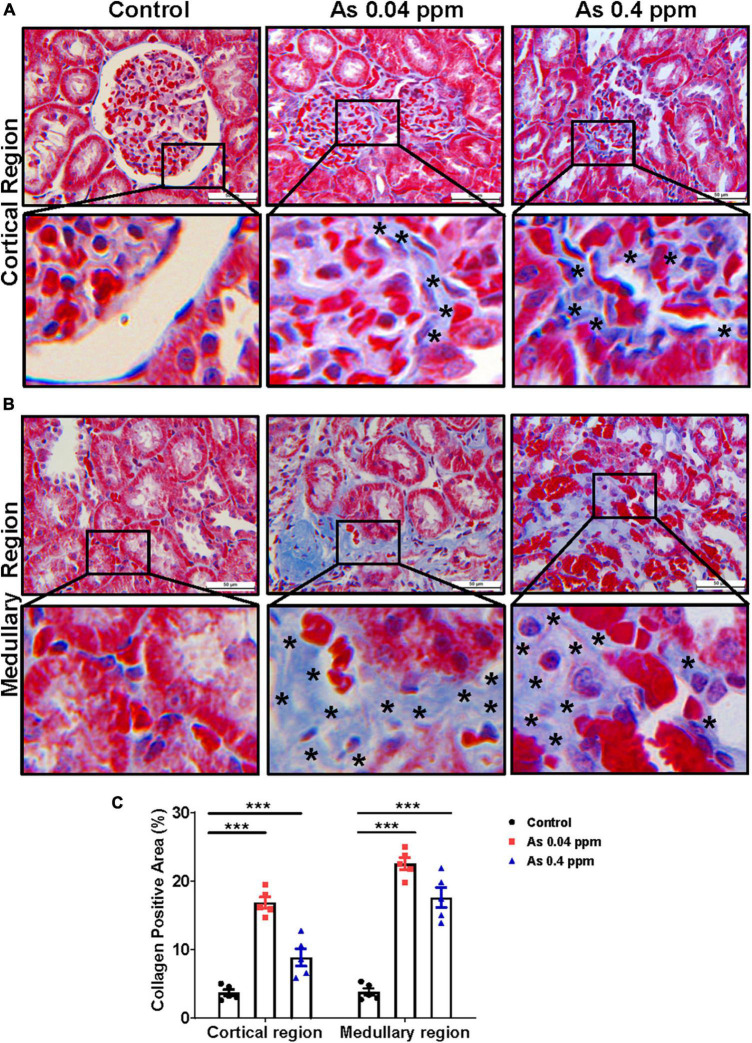
Prenatal As exposure promotes collagen deposition in the kidney. Kidney sections of exposed animals were stained with trichrome staining at 38 weeks of age. The blue stained regions represent the collagen. The level of collagen is significantly higher around the glomerular region and their enlarged inset view shows the specific site of collagen deposition **(A)**. Collagen deposition was also prominent in the medullary region. The specific site of collagen deposition is shown in the inset view **(B)**. The asterisk shows the site of collagen deposition. The level of collagen in the glomerular and medullary region **(C)** is graphically represented. Bar = 50 μm. ***p < 0.0001.

Collagens and other matrix proteins, such as fibronectin and various proteoglycans, cumulatively compose the interstitial matrix of the kidney (60). Injured tubules are known to release excess extracellular matrix components responsible for tissue fibrosis. Trichrome staining of the kidney for extracellular matrix (ECM) components showed a significant increase in collagen deposition in prenatally As exposed groups in the cortical glomerular region (Figure 9A) and in medullary regions of the kidney (Figure 9B). Analysis by Image J software also showed up to a 3-fold increase in collagen content in animals exposed to 0.04 ppm As compared to control in cortical glomerular and medullary regions of the kidney (Figure 9C).

**FIGURE 10:**
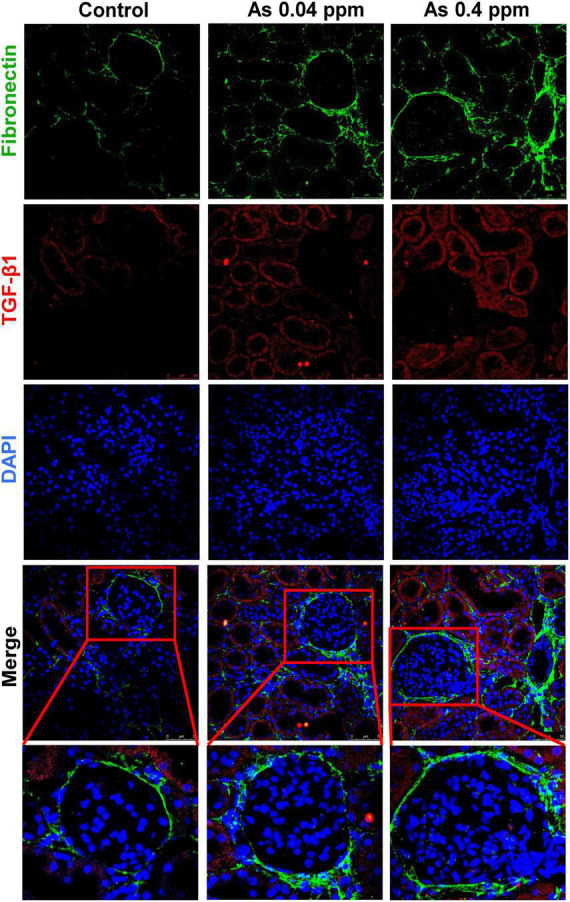
Collagen deposition corresponds with increased fibronectin expression in the cortical region of the kidney. Kidney sections of exposed animals were stained at 38 weeks of age. Increased fibronectin and TGF-β1 expression were observed in the cortical region. Bar = 25 μm.

**FIGURE 11:**
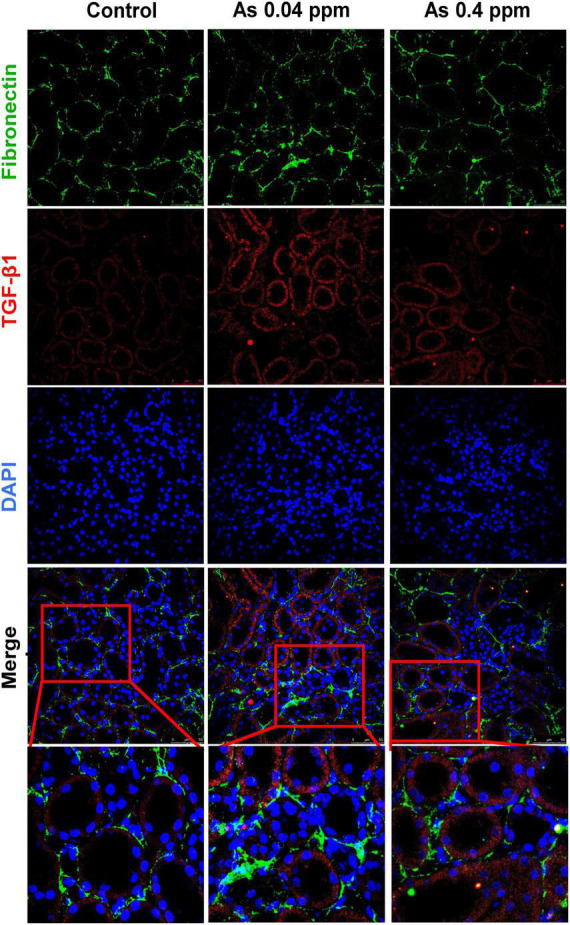
Collagen deposition corresponds with increased fibronectin expression in the medullary region of the kidney. Kidney sections of exposed animals were stained at 38 weeks of age. Increased fibronectin and TGF-β1 expression were observed in the medullary region. Bar = 25 μm.

Furthermore, the level of fibronectin, a potential biomarker of fibrosis, was assessed by immunohistochemistry. Fibronectin, a matrix protein, showed higher expression in As-exposed progeny both in the cortical glomerular region (Figure 10) and in the medullary regions of the kidney (Figure 11). Fibronectin was highly concentrated around the glomerular region (Figure 10; Supplementary Figures 5A, B) and in the interstitium of the tubular region (Figure 11; Supplementary Figures 5C, D).

### Alteration in the TGF-β1 expression and its regulation

**FIGURE 12:**
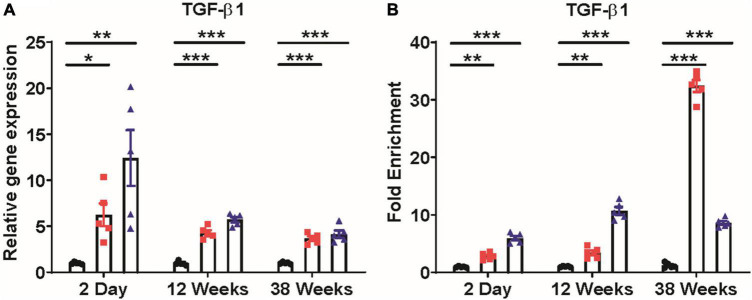
Expression of TGF-β1 and DNA methylation alterations in TGF-β1 gene-body. Prenatal As exposure induces TGF-β1 expression in the kidney **(A)**. Change in TGF-β1 gene-body methylation in exposed progeny was studied through methylated DNA immunoprecipitation (MEDIP) assay. Hypermethylation of the TGF-β1 gene-body was shown and represented as fold enrichment over controls **(B)**. The data represent the mean ± SEM, *n* = 5 (number of experimental sets). *p < 0.05, **p < 0.001, and ***p < 0.0001.

TGF-β1, a potent profibrotic biomarker, was highly upregulated in kidneys at the mRNA level. A significant increase in TGF-β1 expression at mRNA level was observed in kidneys of 2-day (*p* ≤ 0.05, *p* < 0.001), 12 weeks (*p* ≤ 0.0001, *p* ≤ 0.0001), and 38 weeks (*p* ≤ 0.0001, *p* ≤ 0.001) old 0.04 ppm and 0.4 ppm prenatally exposed groups, respectively (Figure 12A). As TGF-β1 was one of the primary cytokines in our study, which is associated with renal fibrosis, we examined the DNA methylation status of the TGF-β1 gene-body region by MeDIP assay. Methylation patterns were assessed at 2 days, 12- and 38-week time points (Figure 12B). Dose-dependent hypermethylation was observed in the gene-body region for prenatally exposed groups at all time points. However, at 38 weeks’ lower dose, i.e., 0.04 ppm showed the highest levels of hypermethylation (Figure 12B).

## Discussion

The developmental origin of health and disease (DoHAD) hypothesis suggests that early-life exposure to stress could have lifelong adverse health effects on the progeny (61). Several studies now focus on early life toxicant exposure (62) on generation and transgenerational changes in the offspring. As is a known endocrine-disrupting chemical that disrupts nuclear receptor and hormonal signaling pathways (63–65) and also can cross the transplacental barrier, thereby having the potential to cause detrimental developmental changes during gestation period which is a highly vulnerable period for both the mother and fetus. The developmental stage represents a highly rapid phase of organ development and growth (66). Epidemiological studies on the Chilean population suggest early life sensitivity to As-induced carcinogenicity. Studies showed an increased rate of mortality due to multiple organ cancers, including renal and bladder cancers, in young adults exposed to As through drinking water (0.85 ppm) in the early stages of life (9, 24, 67). Transitional cell carcinoma of the renal pelvis and ureter and renal cell carcinoma were common in the Chilean population, with increased risks manifesting 40 years after As exposure was reduced (68).

As the prenatal period is known to be the highly vulnerable stage, the present study aimed to identify the effect of prenatal As exposure on risk factors that promote CKD, a less studied aspect of As exposure. Our current study is in continuation with our previous study, where we showed a dose-dependent increase in KIM-1 and Cinc-1 (IL-8 analog) expression in 2-day old prenatally As exposed pups (0.1 and 4 ppm) (41). Previous studies have also observed that prenatal exposure to As at high doses (50 ppm) could promote renal injury (47). In the present study, the animals were subjected to pre-gestational 15 days As exposure (0.04 and 0.4 ppm), which continued during mating and until the gestational period.

During the initial stages of the experiment, various CKD-promoting factors such as altered cytokine levels in the blood plasma and impaired glucose metabolism were assessed. The study showed a significant increase in TGF-β1, IL-1α, MIP-2α, MIP-3α, RANTES, TNF-α, and VEGF levels in the blood plasma in 2-day-old prenatally exposed pups, which persisted till 38-weeks of age (Figure 1).

Previous studies showed upregulation of RANTES in the renal tubular epithelium (69) and TNF-α in mouse mesangial cells (59, 70, 71). In addition, an elevated level of TNF-α has also been associated with loss of renal function (72).

Many of the identified cytokines are associated with obesity and metabolic changes (73). The elevated expression level of TGF-β1 and TNF-α has a strong correlation with the pathogenesis of type I diabetes (52, 53, 74). In our study, a persistent increase in TGF-β1 and proinflammatory cytokines accompanied by increased blood glucose levels might lead to progressive loss of kidney function in prenatally exposed rat offspring. Increased Tgf-β1 expression at mRNA level was also observed in the kidneys of exposed male progeny at 2-day, 12- and 38 weeks of age (Figure 12A). In addition, an increase in Tgf-β1 expression in kidneys was correlated with the level of extracellular matrix deposition (Figures 9–11).

Altered expression of metabolic factors has been linked with weight gain and loss of glucose homeostasis (73, 75). Glucagon levels were lower, while Leptin and Ghrelin levels were persistently elevated (Figures 2E, F). Glucagon is released by alpha cells of islets when there is decreased blood glucose level. It is counterbalanced by insulin secretion. Leptin is an adipocytokine associated with satiety signaling and is reported to be increased during obesity (73) without any beneficial effect on energy homeostasis, suggesting leptin resistance (76). Ghrelin is a hunger hormone produced by the gastrointestinal (GI) tract and is known to increase appetite. Elevated Leptin and Ghrelin may also inhibit insulin production from beta cells, thereby preventing efficient glucose metabolism, as suggested by some earlier studies (77, 78). Dysregulated expressions of glucagon, leptin, and ghrelin (Figures 2E, F) may have contributed to weight gain (Figure 2A; Supplementary Figure 3) and altered glucose metabolism (Figures 2B–D) observed in gestationally As exposed adult offspring. Altered OGTT (Figure 2C) but normal ITT (Figure 2D) suggests that while animals had impaired glucose tolerance (glucose intolerance), their insulin responsiveness was not affected. Previous studies also showed that perinatal As exposure induces glucose intolerance in the offspring (79). Prenatal As exposure may affect glucose homeostasis by altering its uptake, transport, or metabolism, which may further lead to defects in ATP-mediated insulin release, thereby limiting cellular response to elevated glucose levels (80–82).

As prenatal As exposure induced elevated cytokine and adipokine levels in the blood plasma and impaired glucose metabolism is associated with kidney damage, we assessed the levels of nephrotoxic biomarker, histopathological and ultrastructural alterations at the cellular and subcellular levels. In the present study, nephropathy was observed in prenatally exposed adult offspring as assessed through histopathological parameters at 38 weeks of age (Figures 4D, E, 9). The renal injury was corroborated by an increase in early and late nephrotoxicity markers such as kidney injury molecule-1 (KIM-1) (Figure 4C), total protein level in urine (Figure 4B) and plasma creatinine level (Figure 4A) at 12- and 38-weeks of age. KIM-1 level was also significantly higher in the kidney lysate of 2-day-old prenatally exposed pups.

Kidney damage was characterized by increased deposition of ECM proteins (Figure 9), including fibronectin (Figures 10, 11), and the glomerular region showed signs of diabetes-associated injury, including mesangial expansion, increased cellularity, and closed capillaries (Figures 4D, F, G, 9). These histopathological changes were supported by ultrastructural changes as observed by TEM, such as damaged glomerular podocytes (Figures 5B, D) and mitochondria in PCT (Figure 7) and DCT (Figure 8) in the prenatally exposed 38-week-old offspring. Moreover, increased accumulation of PAS-positive material in the glomerulus of the As exposed groups (Figures 4E, H) suggested increased deposition of glucose-rich moieties in the kidney, which is often associated with high blood sugar levels.

Some epidemiological studies show that As exposure has been associated with CKD (32, 34, 36, 83–87). However, most of these studies do not explain the mechanistic basis or clarify whether the effects are a combination of gestational and adult exposure, which is difficult to delineate in population studies. Studies done on people born during the Dutch famine and conditions of starvation in Bangladesh have shown that stress during the gestation period could have lifelong effects on the health of the progeny and lead to changes in the methylome and metabolic disorders (88–94). Prenatal As exposure is known to alter DNA methylation patterns and may also modulate the expression of various proinflammatory and development-related genes (19, 95–97). As the detrimental changes in our experiments were observed just after birth (2 days), we assessed whether epigenetic changes during fetal development contributed to persistent changes in implicated genes even after 38 weeks of exposure. The expression of all three major Dnmts, i.e., Dnmt-1, Dnmt-3a, and Dnmt-3b, was high from 2-day to 38 weeks of age in As exposed groups (Supplementary Figure 4). Prenatal As exposure also induced hypermethylation of TGF-β1 gene-body (Figure 12B), which correlated with increased TGF-β1 gene expression (Figure 12A) in kidneys of prenatally As exposed rats. Some earlier studies have shown a positive correlation between gene-body hypermethylation and gene expression (98–100).

The non-monotonous dose-response (NMDR) of some of the variables was an intriguing aspect observed in the current study. The effects of iAs on physiological parameters may be the cause of NMDR, exemplifying the complex interactions between an environmental factor and the physiology and metabolism of the exposed individual. These physiological parameters include iAs’ complex interaction with nuclear and hormone receptors, which further disrupts nuclear and hormonal signaling (26, 28, 29, 101). For instance, As has been shown to modulate the glucocorticoid receptor pathway, alter steroid signaling, and affect the thyroid, adrenal, and gonadal endocrine systems (102, 103). The interaction of iAs with hormone and nuclear receptors is complicated further by gestational exposure and its biotransformation potential (104). Multiple studies have linked NMDR and endocrine disruption (105), with different profiles including the most common inverted U shape, with the response at intermediate dose and no or low response at high and low doses. In our recently published study, we found a similar NMDR in terms of aggravated carcinogenesis in a mouse model system at similar doses (106).

**FIGURE 13:**
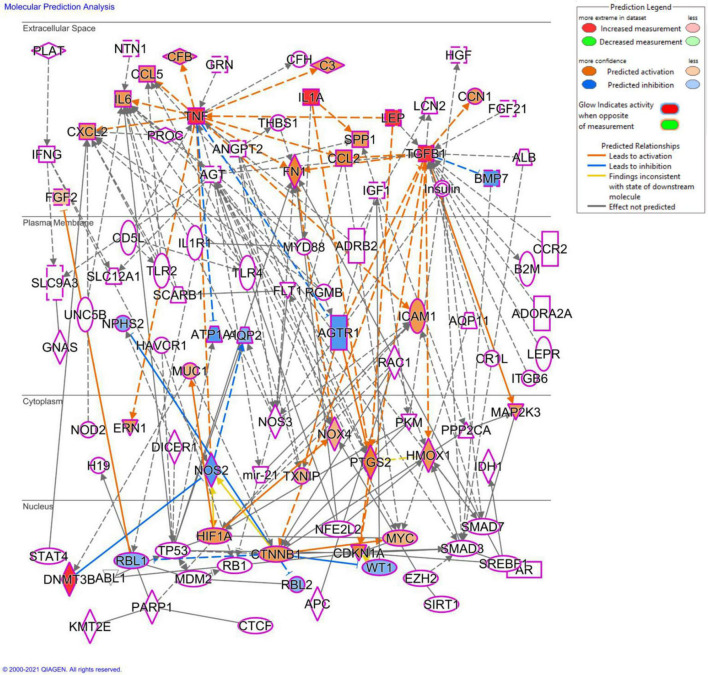
Molecular prediction analysis using QIAGEN ingenuity pathway analysis (IPA) software. The molecular prediction tool shows the plausible mechanisms associated with the differentially expressed genes and cytokine, which will be used in our further study to determine the mechanistic basis of prenatal As exposure induced renal injury.

In the present study, kidneys seem to be the highly vulnerable organ to prenatal As exposure in males *via* increasing the production of profibrotic and adipogenic mediators. Furthermore, prenatal As exposure may cause persistent inflammation and metabolic impairment in male offspring *via* altered DNA methylation patterns, the significance of which needs to be determined in future studies that will also focus on sex-specific differences. Molecular prediction analysis of differentially expressed cytokines and genes using QIAGEN Ingenuity Pathway Analysis (IPA) will provide further insights into studying detailed potential signaling pathways involved in prenatal As exposure-induced CKD progression (Figure 13).

## Conclusion

Our study provides evidence that prenatal stress to very low doses of As could be sufficient to promote the early onset of chronic kidney disease in males. The onset of renal injury following prenatal As exposure is strongly linked with a persistent increase in the expression of proinflammatory, profibrotic cytokines, and adipokines, leading to progressive loss of renal function in male progeny. The kidney seems to be the primary tissue modulated by prenatal As exposure. The study highlights *in utero* stress due to As exposure as the major causal factor responsible for the manifestation of As-associated kidney and associated disorders in males using an animal model, which may have relevance for the human population living in As endemic areas. It may be helpful to monitor people who were born in As endemic areas but have since moved to the unaffected region, as they may still have a higher susceptibility to early onset of adult disorders.

## Data availability statement

The original contributions presented in this study are included in this article/Supplementary material, further inquiries can be directed to the corresponding authors.

## Ethics statement

The animal study was reviewed and approved by Institutional Animal Ethics Committee (IAEC) of CSIR-Indian Institute of Toxicology Research, India.

## Author contributions

RS and RT made a substantial contribution to the conception and design of the experiment, acquisition, analysis, and interpretation of data, and drafting of the manuscript. VSh and SSi performed the MeDIP assay and did all statistical analyses. VSh, HK, and SG participated in revising the article critically for important intellectual content. KK, SSh, and NA performed TEM imaging and analysis. KM and PJ contributed to sample preparation. DS provided animals and associated technical support. SP and SR gave technical support and conceptual advice. AG and SG contributed to human sample collection. SG and KK contributed to sample processing. VSr planned and supervised the project and reviewed the manuscript. All authors contributed to the article and approved the submitted version.
