# Supplementary material for: Prenatal arsenic exposure induces immunometabolic alteration and renal injury in rats
Source: Front Med (Lausanne). 2023 Jan 11;9:1045692. doi: 10.3389/fmed.2022.1045692 (PMC9874122; doi:10.3389/fmed.2022.1045692)
Supplement: Supplementary file 1 [file Data_Sheet_1.docx]

**Fig. S1. Sex ratio of pups (male fraction) born to mothers exposed to As was non-significant as compared to the non-As treated control group.** The data represents the mean ±SEM, n = 5 (number of experimental sets).

**Fig. S2. Prenatal As exposure promotes expression of cytokines in female offspring.** Prenatal As exposure induces expression of cytokines in the female progeny born. Level of cytokines was assayed in blood plasma at 12- and 38-weeks. The data is represented as Mean fluorescence intensity (MFI). The data represents the mean ±SEM, n = 5 (number of experimental sets). *p < 0.05, **p < 0.001 and ***p<0.0001.

**Fig. S3: Average body weight of prenatally exposed rats.** The data represents the mean ±SEM, n = 5 (number of experimental sets). *p < 0.05, **p < 0.001 and ***p<0.0001.


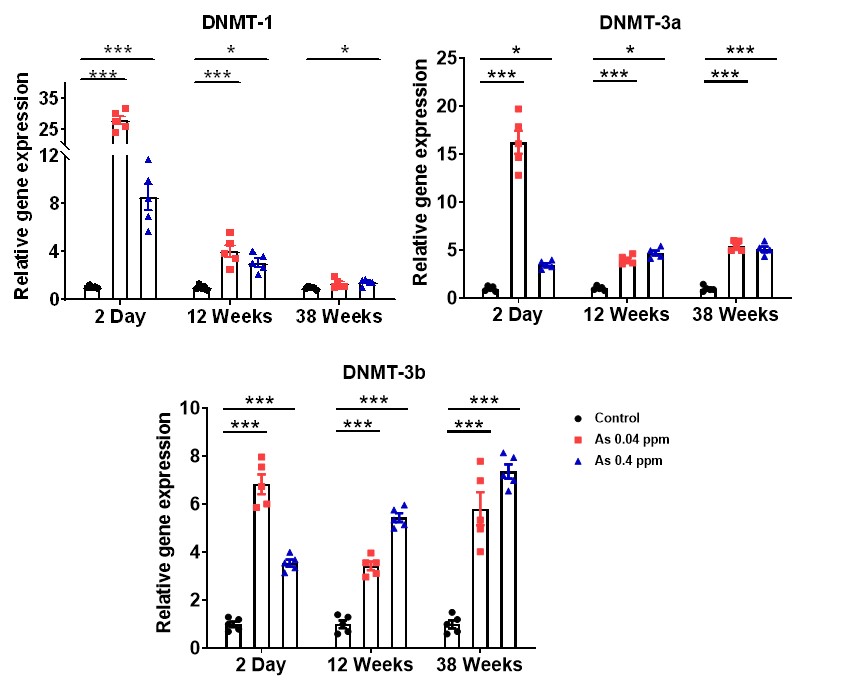


**Fig S4. Prenatal arsenic exposure leads to altered expression of DNA methyltransferases.** Prenatal arsenic exposure leads to increased expression of DNMT-1, DNMT-3a and DNMT-3b at different age (2 day, 12 and 38 weeks). The data represents mean ±SEM, n = 5 (number of experimental sets). *p < 0.05, and ***p<0.0001.

**Supplementary Table S1:** List of real time PCR primers

| **Gene** | **Forward** | **Reverse** |
| --- | --- | --- |
| *β-actin* | GATCAAGATCATTGCTCCTCCTGA | CAGCTCAGTAACAGTCCGCCT |
| *18s* | AAACGGCTACCACATCCAAG | CAATTACAGGGCCTCGAAAG |
| *Dnmt-1* | GGAGCCCAGCCCAGAGTATG | TGGGCGTCTCATCATCGTCC |
| *Dnmt-3a* | GGCTGCACCTGGCCTTATG | ACAGTGGGAGATGCAGGGTC |
| *Dnmt-3b* | CCCAAGGCGTATTCGTCGCC | GCCGCGCTACGTTTACTTGG |
| *Tgf-β1* | GGACCGCAACAACGCAATCT | AAAGACAGCCACTCAGGCGT |

**Supplementary Table S2:** The coefficient of variation (%) of the accessed analytes was determined using Graphpad Prism.

| Analytes | Groups | 2 Day | 12 weeks | 38 weeks |
| --- | --- | --- | --- | --- |
| TNF-α | Control | 29.85 | 13.33 | 19.57 |
|  | 0.04 ppm | 15.9 | 13.06 | 20.45 |
|  | 0.4 ppm | 14.3 | 13.51 | 18.91 |
| TGF-β1 | Control | 16.72 | 13.95 | 19.35 |
|  | 0.04 ppm | 17.04 | 19.2 | 18.95 |
|  | 0.4 ppm | 17.99 | 8.669 | 11.75 |
| IL-1α | Control | 26.98 | 17.7 | 10.49 |
|  | 0.04 ppm | 13.36 | 21.21 | 12.55 |
|  | 0.4 ppm | 17.11 | 17.44 | 15.18 |
| MIP-2α | Control | 19.83 | 16.3 | 23.41 |
|  | 0.04 ppm | 13.62 | 17.14 | 21.07 |
|  | 0.4 ppm | 18.39 | 13.25 | 18.8 |
| MIP-3α | Control | 24.58 | 16.37 | 29.46 |
|  | 0.04 ppm | 17.25 | 16.96 | 16.37 |
|  | 0.4 ppm | 18.83 | 16.67 | 21.97 |
| RANTES | Control | 13.65 | 20.07 | 44.63 |
|  | 0.04 ppm | 15.38 | 15.16 | 18.84 |
|  | 0.4 ppm | 11.95 | 13.09 | 18.76 |
| VEGF | Control | 24.52 | 19.2 | 24.45 |
|  | 0.04 ppm | 25.07 | 12.2 | 17.06 |
|  | 0.4 ppm | 19.83 | 13.65 | 12.51 |


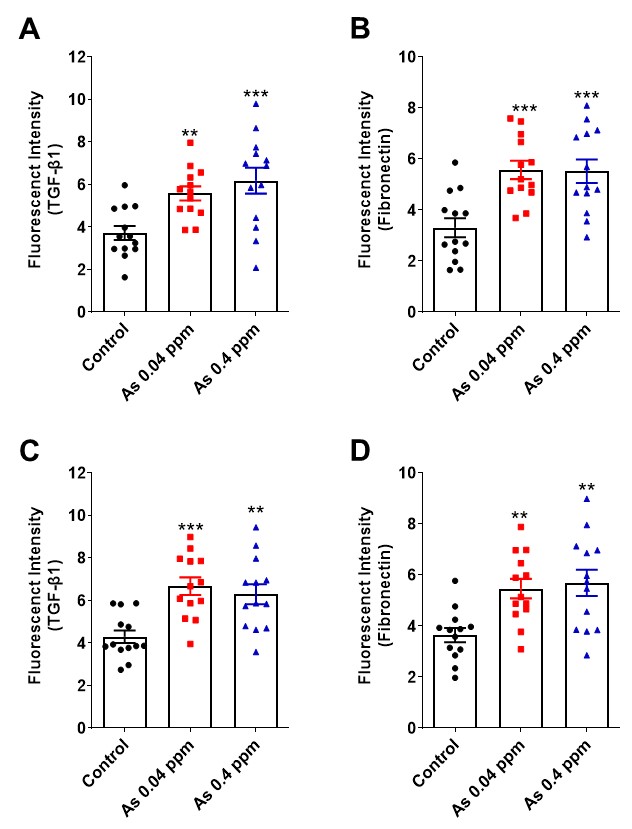


**Fig S5. IHC Image analysis using image showed a significant increase in TGF-β1 and fibronectin expression level in prenatal arsenic exposed male rats.** The data represents mean ±SEM, n = 5 (number of experimental sets). *p < 0.05, **p < 0.001 and ***p<0.0001.

**Fig S6. A significant decrease in the glucagon level in pancreatic tissue lysate was observed in prenatal arsenic exposed male rats.** The data represents mean ±SEM, n = 5 (number of experimental sets). **p < 0.001 and ***p<0.0001.
